# Advances in novel therapeutic approaches for periodontal diseases

**DOI:** 10.1186/s12903-022-02530-6

**Published:** 2022-11-15

**Authors:** Md Mahamudul Haque, Katherine Yerex, Anastasia Kelekis-Cholakis, Kangmin Duan

**Affiliations:** 1 Department of Oral Biology, University of Manitoba, Winnipeg, MB Canada; 2 School of Dental Hygiene, University of Manitoba, Winnipeg, MB Canada; 3 Department of Dental Diagnostic and Surgical Sciences, Rady Faculty of Health Sciences, Dr. Gerald Niznick College of Dentistry, University of Manitoba, Winnipeg, MB Canada; 4 Children’s Hospital Research Institute of Manitoba (CHRIM), Winnipeg, MB Canada

**Keywords:** Periodontal diseases, Biofilms, Antibiotic resistance, Quorum sensing inhibitors, Anti-virulence, Immune modulators, Novel therapeutic strategies

## Abstract

Periodontal diseases are pathological processes resulting from infections and inflammation affecting the periodontium or the tissue surrounding and supporting the teeth. Pathogenic bacteria living in complex biofilms initiate and perpetuate this disease in susceptible hosts. In some cases, broad-spectrum antibiotic therapy has been a treatment of choice to control bacterial infection. However, increasing antibiotic resistance among periodontal pathogens has become a significant challenge when treating periodontal diseases. Thanks to the improved understanding of the pathogenesis of periodontal disease, which involves the host immune response, and the importance of the human microbiome, the primary goal of periodontal therapy has shifted, in recent years, to the restoration of homeostasis in oral microbiota and its harmonious balance with the host periodontal tissues. This shift in therapeutic goals and the drug resistance challenge call for alternative approaches to antibiotic therapy that indiscriminately eliminate harmful or beneficial bacteria. In this review, we summarize the recent advancement of alternative methods and new compounds that offer promising potential for the treatment and prevention of periodontal disease. Agents that target biofilm formation, bacterial quorum-sensing systems and other virulence factors have been reviewed. New and exciting microbiome approaches, such as oral microbiota replacement therapy and probiotic therapy for periodontal disease, are also discussed.

## Background

**Fig. 1:**
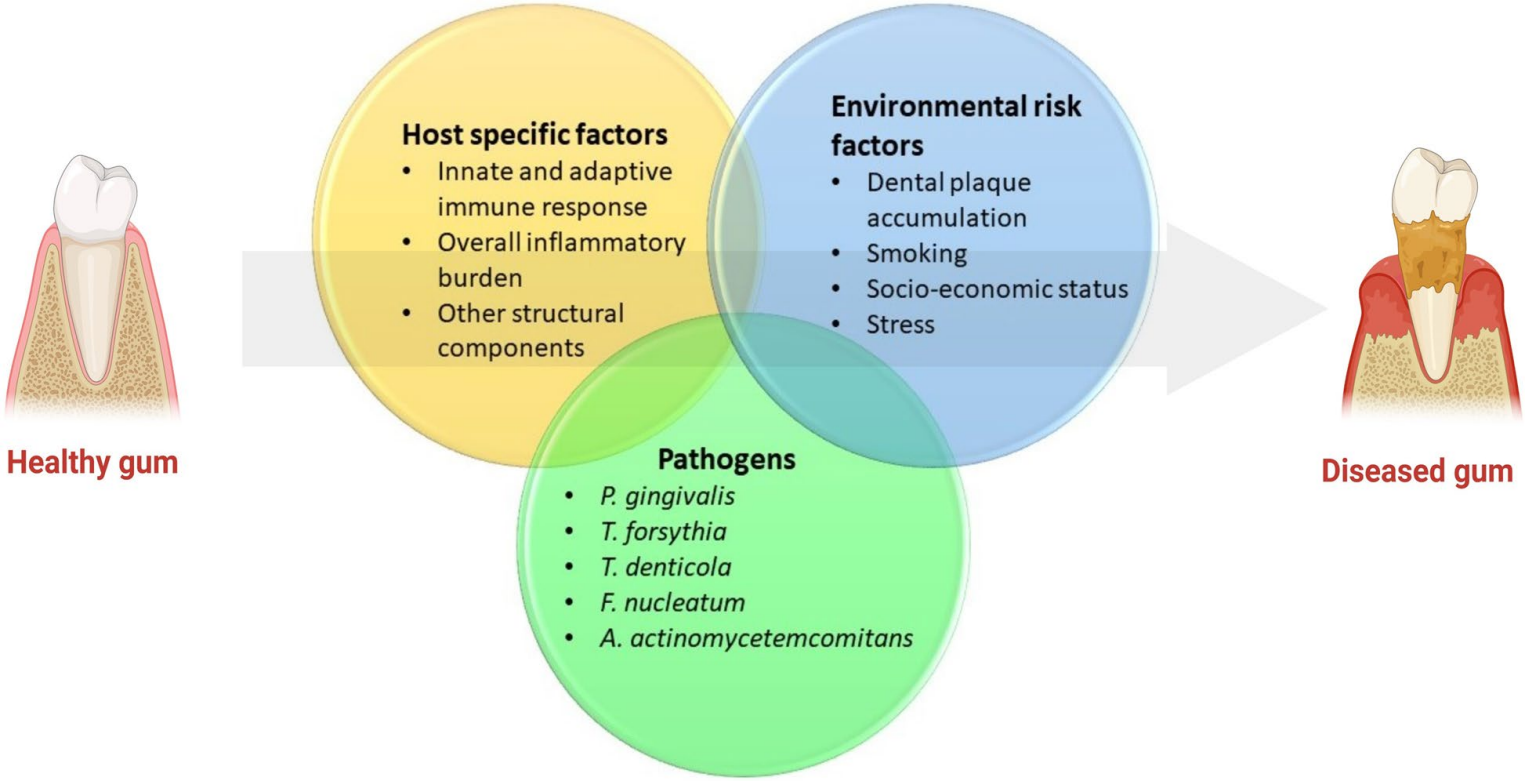
Overview of periodontal disease and factor that affects susceptibility to periodontal infections**.** Periodontal pathogens use an array of virulence factors to initiate periodontal disease. These virulence factors help the pathogens escape host cells' immune response. The progression of periodontal disease depends on the host’s response to microbial dysbiosis in biofilms. The severity and extent of periodontal disease are also influenced by host-specific, genetic, and environmental factors [7]

Periodontal disease is one of the most common chronic inflammatory diseases in humans, affecting up to 90% of the global population [1, 2]. It is the pathologic process affecting the periodontium. According to the American Academy of Periodontology (AAP) and European Federation of Periodontology (EFP) guidelines, periodontal diseases and conditions are classified into the following categories: periodontal health, gingival diseases, and conditions; periodontitis; and other conditions affecting the periodontium. Based on the severity and complexity, periodontal disease can be further classified into stage I-IV [3, 4]. The most common forms of periodontal disease are gingivitis and periodontitis. These conditions involve a group of oral pathogens that can form biofilms on the tooth's surface and in the periodontal pocket and elicit a host inflammatory response. Dental biofilm-induced gingivitis involves the accumulation of dental biofilm and is characterized clinically by swelling, redness, and tissue bleeding [5]. In patients with gingivitis, the alveolar bone and the periodontal ligaments are usually not damaged [6]. In contrast, periodontitis is characterized by the destruction of alveolar bone and periodontal ligaments [5]. The clinical manifestations of periodontitis involve the periodontal pocket, an ideal place for the colonization of bacteria [7]. The primary oral pathogens that contribute to the initiation and progression of periodontitis include *Porphyromonas gingivalis*, *Tannerella forsythia* and *Treponema denticola.* These bacteria are known as the red complex as they are found together in periodontal pockets where extensive periodontal tissue damage is evident [8]. Other periodontitis-associated bacteria include *Fusobacterium nucleatum*, *Aggregatibacter actinomycetemcomitans,* and *Prevotella intermedia* [9]. In addition to pathogenic bacteria, the disease progression is influenced by host-specific and environmental risk factors (Fig. 1).

Physical removal of the biofilms and calculus by scaling has been the most widely used clinical option in periodontal disease treatment [10]. While dental biofilm-induced gingivitis can be reversed by improved oral hygiene and supragingival scaling, surgical periodontal procedures are performed with more advanced periodontal disease. Antimicrobial agents such as chlorhexidine and systemically administered antibiotics are sometimes used in conjunction with surgical treatments for periodontal diseases. The most commonly used systemic antibiotics include amoxicillin or metronidazole and extended-release doxycycline. Antibiotics are often given to patients for prophylactic purposes following invasive periodontal surgeries. However, in alignment with the current trend of increasing antimicrobial resistance in human pathogens, antibiotic resistance has increased among patients with periodontal disease in recent years [11]. The unique periodontal environment and biofilm formation make these bacteria less susceptible to antibiotics [12]. New therapeutic strategies are needed for periodontal diseases.

**Fig. 2:**
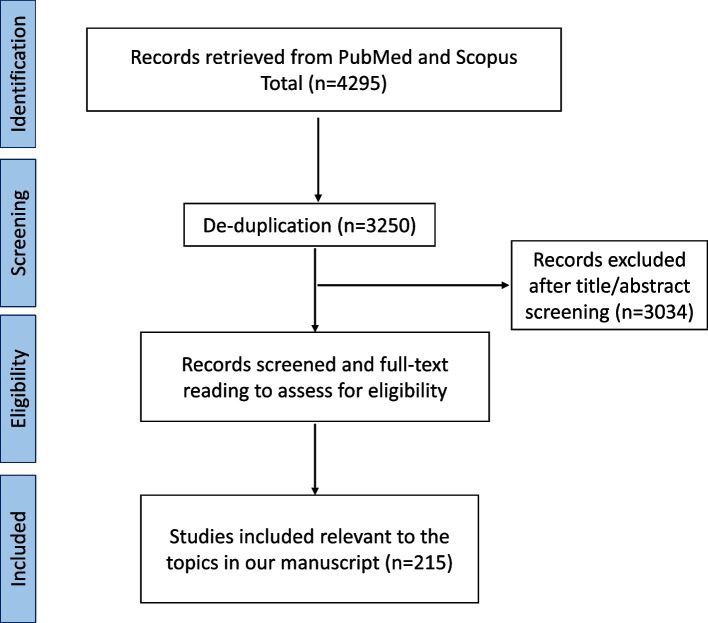
Flowchart of the literature search strategy and selection criteria. The literatures were retrieved from two databases: PubMed and Scopus. Search terms used in two separate searches includes: 1. (periodontal) AND (antibiotic) OR (virulence) OR (biofilm) OR (immunomodulatory AND therapy) OR (probiotic) OR (quorum AND sensing); 2. (periodontal* AND disease) AND (therapeutic*) AND (antibiotics OR antimicrobials) AND NOT (regenerative) AND NOT (rat or animal) AND NOT (case AND reports). The literatures were limited to articles published in English and within 15 years (2008–2022), while those of animal studies, case studies, or presented in the form of abstracts were excluded. 215 references relevant to the topics were cited in this review

Several new therapeutic and preventive periodontal disease approaches have emerged in recent years due to the advancement in understanding bacterial pathogenesis, the human microbiome, and host-microbe interaction. Anti-virulence therapy combats against periodontal pathogens by neutralizing their virulence properties, representing a promising alternative to antibiotic treatment [13]. Host immune modulation by phytocompounds and microbiome-based approaches, such as oral microbiota replacement, are exciting new developments for periodontal disease treatment and prevention. This review discusses the challenge of antibiotic resistance and alternative strategies for periodontal disease treatment. Although this is not a systemic review, we have listed the literature search strategy in Fig. 2.

## Antibiotic therapy and drug resistance in periodontal bacteria

Antibiotics are used to treat infections in the oral cavity and are given in certain circumstances for prophylactic purposes during intraoral procedures where bacteremia is expected to occur [14]. The commonly used local and systemic antibiotics to treat periodontitis are listed in Tables 1 and 2. Local delivery means that the antibiotics are placed directly in the diseased periodontal pocket, and antibiotics for systemic delivery are usually administrated orally.

**Table 1:** List of local antibiotics delivery systems used to treat periodontal patients

**Drugs**	**Polymer Type**		**Outcomes**	**References**
Metronidazole	Polycaprolactone (PCL)	**Nanofiber**	Effective local delivery system of metronidazole in combination with scaling and root planing showed betterment of clinical criteria, for example, plaque index (PI), pocket depth (PD), and gingival index (GI)	[158]
Doxycycline	PCL		Controlled delivery of drug was able to improve PD, PI, and GI effectively in periodontal disease	[159]
Minocycline hydrochloride	Carbopol	**Liposome**	Liposomes containing minocycline hydrochloride showed biocompatibility along with improvement in rat periodontitis	[160]
Minocycline hydrochloride	Poly Ethylene Glycol (PEG)		2% minocycline hydrochloride nanoliposomes strongly inhibited TNF-α secretion by LPS-stimulated macrophages up to 60 h	[161]
Doxycycline	Carbopol		Slow release of drug from nanoliposome gel decreased MMP-8 in rat model of periodontitis	[162]
Metronidazole, Doxycycline	Polymersomes	**Nanoparticle**	Antibiotics encapsulated in polymersomes decreased the number of *P. gingivalis* in monolayer cells as well as in organotypic cultures significantly	[163]
Metronidazole benzoate	Thiolated Chitosan (TCS)- Poly(methacrylic acid) (PMAA)		TCS-PMAA delivery system provided sustained and site-directed release of the drug. Also, the system led to an improvement in oral availability	[164]
Doxycycline	Chitosan		Pre-clinical studies showed that nanoparticles loaded with doxycycline showed entrapment efficacy of 75% and showed antimicrobial activity against *P. gingivalis*	[165]
Metronidazole	Chitosan	**Microparticle**	Hydrogel prepared from chitosan microparticles released metronidazole at an optimal pattern	[166]
hydroxyapatite (HA) and Ofloxacin	Poly Lactic-Co-glycolic acid (PDLGA)		PDLGA microspheres were shown to be biocompatible and porous in nature. This system delivered the drug optimally against *E. coli* and *S. aureus*	[167]
Minocycline hydrochloride	Chitosan		Use of microsphere containing minocycline hydrochloride resulted in the decrease of PDs at 6 months. In addition, bleeding on probing was also decreased significantly in patients	[168]
Doxycycline	Gelatin		Local delivery of antibiotics in the periodontal pock*et al*ong with scaling and root planing led to decrease in PDs. Moreover, the treatment method reduced the number of *P. gingivalis* significantly	[169]
Azithromycin	PLGA	**Gel**	Site-directed delivery of 0.5% of azithromycin showed better clinical output in periodontitis patients	[170]
Metronidazole	Poly-gamma-glutamic acid		Hydrogel was prepared from a non-toxic and biodegradable material such as poly-gamma-glutamic acid. This hydrogel system could be polymerized by light and showed remarkable swelling ability along with a controlled manner of drug release	[171]
Clarithromycin	Carboxy methylcellulose		In adjunction with scaling and root planing treatment, local delivery of 0.5% clarithromycin showed better clinical outputs at 6 months in smokers	[172]
Moxifloxacin	Pluronic Chitosan		Reduction in PD was observed in patients who received moxifloxacin gels at 3 months. In addition, these gels were safe to use and significantly reduced the load of *P. gingivalis* among the periodontal pathogens Shows efficacy against *A. actinomycetemcomitans*	[173] [174, 175]
Chlorhexidine	Chitosan, beta-glycerophosphate		Nontoxic hydrogel system carrying 0.1% chlorhexidine effectively reduced the number of oral pathogens	[162]
Doxycycline	Hydroxyapatite	**Scaffolds**	Scaffolds loaded with doxycycline helps in the process of pre-osteoblasts of bone and tissue repairment in periodontal patients	[176, 177]

**Table 2:** List of Systemic antibiotics used to treat periodontal patients

Antibiotic	Dose and Duration	Disease	Findings	Side Effects	References
Amoxicillin + Metronidazole	Metronidazole: 250 mg Amoxicillin: 500 mg for 2 years	Stage II periodontitis	Antibiotic treatment decreased probing depth (PD) and gained clinical attachment Five randomized clinical trials showed improvement in PD and bleeding on probing	One participant experienced nausea and vomiting and one developed candidiasis Few participants observed light pain or a burning sensation following laser treatment. No side-effects were observed due to administration of antimicrobial agents	[178] [179] [180–184]
	Metronidazole: 500 mg Amoxicillin: 375 mg for 6 months	Stage II periodontitis	Antibiotics reduced the count of sites containing PD as well as bleeding on probing. Antibiotics with SRP led to a decrease in the count of *T. forsythia, F. nucleatum*, and *P. gingivalis*	Majority of the side effects were related to gastrointestinal disturbances such as nausea, diarrhea, vomiting as well as stomach burning	[185, 186]
	Metronidazole: 500 mg Amoxicillin: 500 mg for the time period of 6 months	Stage III periodontitis	Treatment reduced the load of pathogens such as *T. forsythia* as well as *T. denticola.* It also decreased the number of PD sites	One patient reported mild intestinal disturbance and one experienced vertigo	[187]
Amoxicillin + Metronidazole + Doxycycline	Metronidazole: 250 mg Amoxicillin: 250 mg Doxycycline: 100 mg for 2 months	Stage III periodontitis	This drug treatment decreased the number of sites with PD	No side effects were reported related to the antibiotic administration	[188]
Amoxicillin + Clavulanic acid	875 mg each 3 months	Stage I periodontitis	No difference was observed in PD between the control and test groups	/	[189]
Doxycycline + Clindamycin + Metronidazole	Doxycycline: 200 mg Metronidazole: 500 mg Clindamycin: 150 mg for 2 years	Stage IV periodontitis	Treatment decreased PD, gained clinical attachment, and reduced the load of *P. gingivalis* and *Actinobacillus* *Actinomycetemcomitans*	/	[190]
Azithromycin	500 mg for 6 months	Stage II periodontitis	It helped to lessen PD and BANA scores	/	[191]
	500 mg for 6 months	Stage III periodontitis	No reduction in microbial load and PD was observed	Participants did not report any adverse effects from the use of antibiotic and placebo	[192]
	500 mg for the time period of 1 year	Stage III periodontitis	Treatment regimen declined PD and periodontal pathogens significantly	Participants did not report any adverse effects from the use of antibiotic and placebo	[193]
	500 mg once a day for 5 days	Stage III periodontitis	Administration of antibiotic failed to improve clinical parameters when compared to scaling and root planning (SRP) alone	One participant experienced nausea after taking antibiotic	[194] [195]
Metronidazole	400 mg thrice a day for 10 days	Moderate to severe periodontitis (Stage III or IV) with Type 2 Diabetes Mellitus	SRP alone improved the periodontitis and glycemic control significantly	/	[196]

Antimicrobial resistance in periodontal bacteria is a growing concern worldwide. Bacterial pathogens become resistant to antibiotics via several central mechanisms, including degrading antibiotics, altering antibiotic targets, and actively effluxing the drugs from the cell [15].

Pathogens become resistant to antibiotics containing β-lactam rings by producing β-lactamases, which can degrade or modify β-lactam antibiotics [16]. Studies have reported β-lactamase production by periodontal pathogens, including *Porphyromonas*, *Prevotella*, and *Fusobacterium* species [17]. For *P. gingivalis,* the β-lactamase (amoxicillin-based) production rate in clinical isolates is around 7.6% [18]. Walker et al. reported that from 406 samples taken from the gingival crevicular fluid of patients with periodontal disease, β -lactamase property was detected in 64% of the periodontal patients [19]. β-lactamases can be found frequently in periodontal pockets of more than 3 mm deep [19]. Feres et al. demonstrated that bacterial species from subgingival plaque showed resistance against metronidazole and amoxicillin [20]. 21.6% of the *P. gingivalis* isolates from patients with periodontitis were reported to be resistant to metronidazole [21]. Different antibiotic-resistant levels in *A. actinomycetemcomitans* have also been reported depending on geographical distributions [22].

The *tet* gene is known to be responsible for tetracycline resistance. Most *tet* genes originated from periodontal pathogens such as *P. gingivalis* and *F. nucleatum* [23]. The percentage of tetracycline-resistant isolates in dental plaque-associated bacteria is high in children from South Asia and Japan [24]. Isolates of *T. denticola* that are resistant to tetracycline have also been reported [25]. Resistance to tetracycline is a frequent co-marker among penicillin-resistant species of oral origin [26]. Resistance to erythromycin usually occurs due to acquiring two significant genes, *ermF* and *erm*. Major periodontal bacteria such as *T. forsythia* and *P. gingivalis* can carry both *emr* and *tet* resistance genes [27]. Clindamycin resistance shows a high prevalence among periodontal isolates of *F. nucleatum* (36%), *P. gingivalis* (23%), and *Prevotella melaninogenica* (22%) in Columbia [22].

Bacteria associated with periodontal disease naturally form biofilms. Biofilm formation also contributes to the resistance of antibiotics to periodontal pathogens. Studies have demonstrated the increased exchange of antibiotic resistance encoding genes in biofilms [28], which causes the spreading of antibiotic resistance among periodontal bacterial species [29]. The biofilms matrix usually comprises extracellular polymeric substances (EPS), which hinders the dissemination of antibiotics into the bacterial biofilms [30]. The organized structure of biofilms and its matrix also confer diffusion–reaction inhibition to antibiotics, which leads to antibiotic tolerance. Sublethal concentrations of antibiotics in biofilms can also lead to the selection of drug resistance [31]. The slow growth of some bacterial cells in biofilms and the presence of persister cells are major contributing factors to the decreased antibiotic susceptibility in biofilms. It has been reported that bacteria cells in a biofilm can be 1000-fold more resistant to antibiotics than planktonic cells [32].

## Novel treatment strategies for periodontal diseases

Coinciding with the increasing antibiotic resistance among periodontal pathogens, the primary goal of periodontal therapy has shifted in recent years to restoring homeostasis in oral microbiota and its harmonious balance with the host periodontal tissue. Such a shift in the treatment goal and the challenge of drug resistance calls for alternative approaches to traditional antibiotic therapy that indiscriminately eliminates resident commensal or pathogenic bacteria.

Periodontal pathogens use virulence factors to initiate periodontal disease and promote its progression. A promising alternative to antibiotic therapy is targeting pathogenic bacteria's virulence factors and their regulatory systems that control pathogenicity [13]. Such an approach aims to inhibit bacterial virulence factors and thus avert the harmful bacterial pathogens render to the host and reduce pathogen-induced host immune response, reducing the damage to the host tissues. Agents with anti-virulence properties could potentially stop the pathogenic activities of periodontal bacteria [33, 34], disarming its ability to cause periodontal diseases. Strategies can also be developed to target biofilms as forming biofilms by periodontal bacteria causes antibiotic resistance and enables them to evade host immune responses [35, 36]. Because host inflammatory response plays a vital role in periodontal disease, modulators of host innate immunity are another promising strategy to address periodontal disease. The novel oral microbiota replacement therapy resulting from recent advancements in human microbiome studies could potentially be an alternative measure for preventing and treating periodontal disease. Probiotics and oral microbiota replacement therapy are substituting resident periodontal bacteria with non-pathogenic bacteria and restoring healthy homeostasis of the oral microbiome [37].

### Virulence factors in periodontal pathogens and anti-virulence agents

**Fig. 3:**
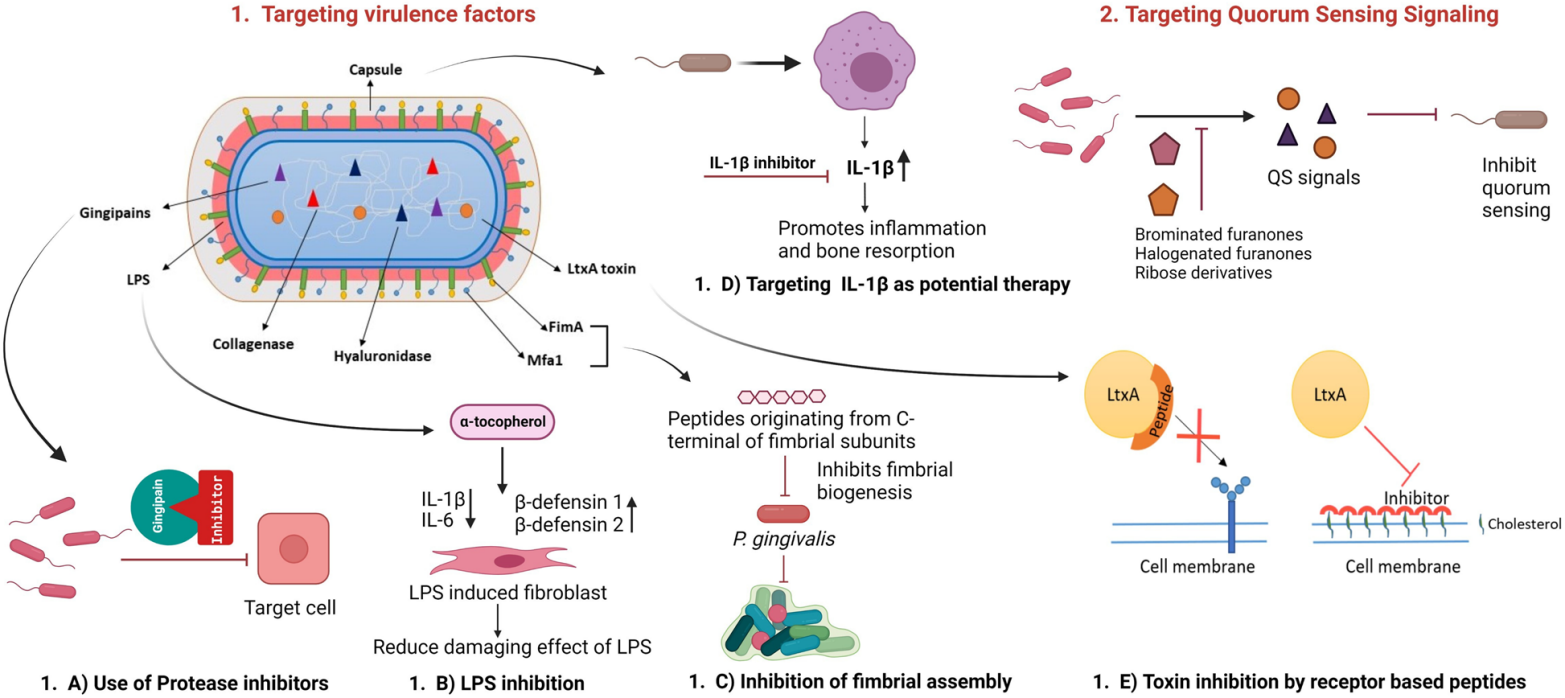
Schematic representation of anti-virulence strategies covered under this review. 1. Targeting virulence factor: (**a**) Protease Inhibitors: Microbial protease plays an essential role in the progression of periodontal diseases and thus could be a potential therapeutic target. The therapeutic approach focused on Gingipain inhibitor (Kgp specific inhibitor A71561) can significantly decrease virulence. (**b**) LPS inhibition: Cell surface components like LPS could be targeted for treating periodontal diseases. α-Tocopherol reduces inflammatory cytokines while increasing antimicrobial peptides, and β-defensins, thereby counteracting the damaging effects of LPS, which play a vital role in the pathogenesis of periodontal diseases. (**c**) Inhibition of fimbrial assembly: Fimbriae are a major structural component of periodontal bacteria. Studies have shown that peptides originating from the conserved C-terminal of FimA and Mfa1 subunits prevent the fimbrial assembly of *P. gingivalis* and interfere with biofilm formation*.* (**d**) Targeting IL-1β as a potential therapy: It has been shown that the capsular polysaccharide upregulates the expression of IL 1-β by activating the JNK pathway in macrophages. Increased IL 1- β levels lead to inflammation and bone resorption, indicating a possible therapeutic strategy to combat this periodontal pathogen. (**e**) Toxin inhibition by receptor-based peptide: *A. actinomycetemcomitans*, associated with periodontal disease, produces leukotoxin (LtxA) during colonization in the host to escape the host immune response. LtxA is the critical virulence factor of *A. actinomycetemcomitans*, which kills leukocytes by recognizing cholesterol and the β2 strands of lymphocyte function-associated antigen-1 (LFA-1) integrin. Molecular inhibitors mimic the target to compete for toxin binding, thus neutralizing toxin binding activity. Small synthetic receptor-specific peptides can hinder LtxA-mediated cytotoxicity by binding to the β domain of transmembrane protein LFA-1. 2. Targeting quorum sensing signalling: QS is the most distributed and studied bacterial communication, which helps periodontal bacteria communicate with each other through signalling molecules and behaviour coordination. Various compounds can block quorum-sensing signals produced by periodontal pathogens, thus inhibiting the disease progression pathway

Virulence factors are either cellular components or secreted products of a pathogen that enable the pathogen to invade the host, evade the host's immune response or cause damage to the host. Several recent therapeutic approaches have been investigated that target critical virulence factors to disrupt the pathogenic processes of periodontal bacteria, which are summarized in Fig. 3.

#### A. Cell surface components as an anti-virulence target against initial infection

Capsules are required for microbial attachment to the host tissue surfaces, marking the first step in bacterial pathogenesis. Co-aggregation of *P. gingivalis* and *F. nucleatum* in the subgingival plaque is mediated by a capsular polysaccharide (CPS) and lipopolysaccharide (LPS) [38]. Increased encapsulation correlates well with increased evasion from phagocytosis and reduced host immune response [39]. The capsular polysaccharide of *Actinobacillus actinomycetemcomitans* upregulates the expression of Interleukin (IL) 1-β by activating the JNK pathway in macrophages. Increased levels of IL 1- β leads to inflammation and bone resorption. Such results support a possible therapeutic strategy to combat this periodontal pathogen by targeting its capsule [40]. IL 1-β could also be targeted for periodontal diseases as it contributes to periodontal pathology and the production of proteinase, which can contribute to the destruction of periodontal tissues. [41].

Natural compounds, alpha-mangostin and grape seed proanthocyanidin extracts, can inhibit the damaging effects of LPS [5]. Apart from that, the soluble film of alpha-mangostin showed antimicrobial and anti-inflammatory activity against periodontal pathogens, especially on *P. gingivalis* [42]. Grape seed contains proanthocyanidin, which helps to reduce the progression of periodontal disease by inhibiting the extracellular collagenase [43]. α-tocopherol reduces inflammatory cytokines while increasing antimicrobial peptides and β-defensins, which can modulate the immune response and counter the detrimental effects of LPS [44] [45]. Tormentic acid can be a potential therapeutic agent against periodontal diseases since it inhibits the production of IL-6 and IL-8 in gingival fibroblasts of humans [5, 46].

Fimbriae are proteinaceous appendages that protrude from the bacterial outer membrane that helps microbes attach to host cell surfaces and other microbial surfaces. *P. gingivalis* possess two antigenically distinct fimbriae subunits, FimA and Mfa1. FimA is found in short fimbriae, and Mfa1 is found in long fimbriae [5]. Immunization with a 43-kDa purified fimbrial protein offers protection against periodontal damage caused by *P. gingivalis* in a germ-free rat model [47]. In a recent study by Alaei et al., peptides originating from the conserved C-terminal of FimA and Mfa1 subunits prevent the fimbrial assembly of *P. gingivalis* and interfer with biofilm formation. The study also showed that treating these peptides reduces *P. gingivalis* adhesion to *Streptococcus gordonii* in a dual-species model [48].

#### B. LtxA disruption

Toxins play an essential role in bacterial pathogenesis. The Gram-negative bacteria *A. actinomycetemcomitans*, associated with periodontal disease, produces leukotoxin (LtxA) during colonization in the host to escape the host immune response [49]. Another toxin these bacteria produce is a cytolethal distending toxin (CDT). Both toxins play a role in compromising the host immune system by targeting different cells [50]. LtxA is the critical virulence factor of *A. actinomycetemcomitans*, which kills leukocytes by recognizing cholesterol and the β_2_ strands of lymphocyte function-associated antigen-1 (LFA-1) integrin [51]. Mechanical removal of plaque and antibiotic treatment, preferably tetracycline, was used. Antibiotics may be considered to treat molar/incisor pattern periodontitis, but only after scaling and root planing have been deemed ineffective. Increased resistance against antibiotics in *A. actinomycetemcomitans* calls for developing alternative treatment methods [52]. It has been reported that small synthetic receptor-specific peptides can hinder LtxA-mediated cytotoxicity by binding to the β domain of transmembrane protein LFA-1 [53]. Studies have found that targeting virulence factors like LtxA could effectively measure against periodontal diseases [54, 55]. Catechins, one class of flavonoids, can inhibit LtxA activity in its soluble form and outer membrane vesicle (OMVs) bound form [56]. At the same time, Chang et al. showed that catechins restructure the soluble form of LtxA, which prevents its interaction with cholesterol on the host cell surface [49].

#### C. Enzymatic virulence factors and their antagonists

Proteases, including gingipains, aminopeptidase IV, and collagenases, play a role in bacterial adhesion, degradation of host proteins (i.e. immunoglobulins), modulating inflammatory responses, and damaging host tissues [57]. Thus, restricting the functionality of these crucial virulence factors will collectively inhibit the microbes' pathogenicity.

##### Microbial proteases

Microbial proteases play a crucial role in the progression of periodontal disease, directly damaging host tissues or indirectly activating a myriad of host proteases and inactivating protease inhibitors in the host [37]. Extracellular proteases can be divided into the following groups: aspartic proteases, cysteine proteases, serine proteases, and metalloproteases [37]. Microbial proteases can serve as important therapeutic targets for treating periodontal diseases. Inhibitors of microbial proteases may prevent infection. These inhibitors can be inorganic or synthetic agents or modified “host products” [37]. Salivary histatin is a peptide with anti-protease activity against many proteases involved in periodontal diseases. Histatin has been delivered subgingivally to treat infections [58, 59]. Other inorganic or synthetic protease inhibitors, including chelators, such as EDTA that inhibit the metalloproteinases, and thiol-blocking agents that inhibit serine and cysteine proteases, are potential agents to inhibit the pathogenicity of periodontal pathogens [37].

##### P. gingivalis gingipains

Members of cysteine proteinases in *P. gingivalis* are called “trypsin-like enzymes” as they cut after the arginine or lysine residue at the C-terminal of polypeptides [5]. These enzymes are collectively known as gingipains, which can be one of two types: 1. Arginine specific that includes RgpA, RgpB, and 2. Lysine specific is known as KgpA [37]. Gingipains hydrolyze a wide range of extracellular and cell-bound proteins encoded by *P. gingivalis* [60].

In a mouse model, treatment of *P. gingivalis* before infection by a Kgp-specific inhibitor (A71561) designed by Curtis et al. decreased the *P. gingivalis* virulence significantly. In contrast, the Rgp-specific inhibitor leupeptin did not influence the pathogenicity [61]. It is stated that leupeptin can attenuate the degradation of an antimicrobial peptide (LL-37), suppress platelets' aggregation, and prevent the inhibition of chemoattractant proteins of monocyte caused by arginine-specific gingipains. The compound further precludes the destruction of collagens and recovers the IL-2 levels [62].

It has been reported that a compound in rhubarb roots named rhein can cause the downregulation of both genes,* rgpA* and *kgp*, in *P. gingivalis* when combined with polyphenols synergistically [63]. Another dual protease inhibitor that is synthetic in nature, KYT-41, can inhibit both *rgpA* and *kgp* proteases and show anti-inflammatory responses [64]. Recent studies have shown that synthetic molecule 2-deoxy-2,3-didehydro-N-acetylneuraminic acid, commonly known as DANA, helps inhibit the growth and biofilm of *P. gingivalis* by decreasing the activity of gingipains in vivo model [65].

The primary component of black tea, theaflavin, has dual effects on gene expression and the catalytic activity of these proteases. Theaflavin not only downregulates the expression of *rgpA* and *kgp* protease genes at sub-MICs but also inhibits the hydrolytic activity of gingipains in a dose-dependent manner [66, 67]. Fractions of cranberry polyphenols inhibited *rgpA* and *kgp* activities in *P. gingivalis* at a concentration of one µg/ml [68]. Macrocarpals found in the leaves of *Eucalyptus globules* inhibited both Arg- and Lys-specific gingipains in *P. gingivalis* in a dose-dependent manner [69].

### Inhibition of Biofilms

Dental biofilm formation in the supragingival and subgingival areas is required to develop dental caries and periodontitis [70]. Bacteria in biofilms exhibit increased resistance to antimicrobial therapy compared to planktonic microbes. Biofilms are hard to treat with the use of antibiotics for several reasons, including decreased penetration of antibiotics to the microbes in the biofilms, increased expression of multiple efflux pumps, reduced growth as well as metabolism, induction of microbial stress response and changes in the expression of outer membrane proteins [70]. Recent randomized clinical trials showed that guided biofilm therapy, together with erythritol powder usage and ultrasonic piezo, is effective against biofilms in periodontal disease [71]. Besides that, several medicinal herbs and herbal compounds have been shown to inhibit biofilm formation and reduce inflammation; therefore, they have been explored as potential therapeutics for oral diseases, including periodontitis [72].

Oxyresveratol or trans-2,4,3′,5′-tetrahydroxystilbene, the main bioactive element of tropical tree *Artocarpus lakoocha,* has been shown to have antioxidant properties [73]. This compound interferes with bacterial cell wall integrity and prevents the growth and biofilm of periodontal pathogen *A. actinomycetemcomitans* [74].

Panduratin A, a natural compound found in *Kaempferia pandurate*, has antibiofilm and bactericidal activities [75]. Epigallocatechin gallate (EGCG), a polyphenol in green tea, has strong anti-oxidative and anti-inflammatory characteristics [76]. This compound is a catechin derivative with a galloyl group attached which interrupts the adhesion of periodontal bacteria to the infection site [77]. Studies showed that epigallocatechin gallate could significantly inhibit the biofilm formation of a significant periodontal pathogen *P. gingivalis* [78].

Cranberry extract containing bioactive compounds such as Licocalchone A and Proanthocyanidins also shows promising benefits against gingivitis by reducing inflammation, biofilm formation and inhibiting proteolytic activity of periodontal pathogens [79, 80]. Pomegranate extract interferes with quorum sensing signalling, which leads to biofilm disruption and impairment of bacteria motility [81]. In addition, pomegranate extract rich in flavonoids reduces inflammation via reducing oxidative stress and interference with NF-κB activity [82].

The effect of the bioactive compounds of plant origin on the growth and biofilm formation of periodontal pathogens and their potential mechanisms of action are summarized in Table **3**.

**Table 3:** Modulatory effects of plant-based compounds on biofilm formation property of periodontal pathogens

Name of plant	Bioactive compounds	Mode of action	Inhibitory effect on the periodontal disease-causing strain	References
*Artocarpus lakoocha* (Moraceae)	Oxyresveratrol	SEM images showed oxyresveratrol might influence bacterial cell wall integrity	*A. lakoocha* extract prevented the growth and biofilm of periodontal pathogen namely *A. actinomycetemcomitans,* in a dose and time-dependent manner	[74]
*Camellia sinensis*	Epigallocatechin gallate (EGCG)	Galloyol moiety ester-linked to 3’-OH of catechin inhibited the adherence factors	Inhibits the growth and biofilm formation of *P. gingivalis*	[78]
*Kaempferia pandurate* (Ginger family)	Panduratin A	Destruction of the cell wall and cytoplasmic membrane	Panduratin A showed MIC of 4 µg/ml against periodontal pathogens (*P. gingivalis* and *P. intermedia*) Panduratin A prevented dental biofilm composed of *S. sanguis*, and *Actinomyces viscosus* by more than 50% at 10 µg/ml following exposure of 15 min	[197] [75]
*Punica granatum* (Pomegranate)	Anthocyanins Caffeic acid Catechin Ellagic acid Epigallocatechin Gallic acid	Flavonoids absorb free radicals to prevent oxidative damage of important biomolecules inhibits NF-κB activity to impart an anti-inflammatory effect	Suppress the growth and biofilm formation of dental pathogens Mouthwash containing pomegranate extract lowered the plaque index of *A. actinomycetemcomitans, P intermedia,* and *P. gingivalis*	[82] [198]
*Vaccinium macrocarpon* (Ericaceae fruit), also known as cranberry extract	Licocalchone A Proanthocyanidins	Inhibition of the enzymes and proteases needed to form biofilms	Cranberry extract can suppress the growth of *F. nucleatum* and *P. gingivalis* Adherence factors are inhibited in case of *P. gingivalis* as well as co-aggregation with other bacteria are suppressed Also inhibit the formation of biofilm by both bacteria at concentrations or greater than 62.5 μg/ml	[80]

### Quorum sensing and quorum quenching in periodontal pathogens

Bacterial communication plays a vital role in establishing a host-infection process successfully [83]. Communication between oral biofilms has been demonstrated in numerous studies. Quorum sensing (QS) is the most distributed and studied bacterial communication, which helps periodontal bacteria communicate with each other through signalling molecules and behaviour coordination [84]. QS gene expression and signalling are mostly limited to periodontal pathogens, including *P. gingivalis* and *A. actinomycetemcomitans* isolated from patients with dental biofilm-associated diseases [85]. *P. gingivalis* uses signalling molecules for interspecies interaction to promote a microbial dysbiosis which leads to periodontal disease [86]. Thus, targeting those signalling pathways serves a new way for preventing periodontal diseases [86]. A study conducted by Azakami et al. reported that the homologous luxS gene (required for the synthesis of autoinducers-2), found in other periodontal pathogens, *P. intermedia*, and *F. nucleatum*, as well as in *Eikenella corrodens,* initiates the formation of biofilms in the oral cavity through LuxS-dependent pathway [87]. *A. actinomycetemcomitans* can also form mature biofilm through autoinducer-2 dependent quorum sensing manner [88]. AHL-mediated QS, like *Aliivibrio fischeri,* has not been reported in periodontal pathogens [85].

Developing a drug that could inhibit quorum sensing has been gaining attention in recent years. Quorum quenching, commonly known as inhibiting the signalling pathway of quorum sensing, can be an attractive solution for targeting periodontal bacteria by destroying bacterial communication, a critical aspect of biofilm formation and virulence factor expression [89, 90]. Plancak et al. have discovered naturally occurring inhibitory compounds and synthetic derivatives, which are classified based on their structural quorum sensing properties, [88]. Studies have shown that *Delisea pulchra*, a marine alga produces various components of vanilla extract, garlic, halogenated furanones and vanilla extract. These compounds have quorum quenching properties that help block the quorum sensing singling in periodontal pathogens [91–94]. It has been reported that adding ribose derivatives to the culture media was shown to negatively affect the formation of biofilms in *A. actinomycetemcomitans* [95].

Similar functional properties are shared by ribose binding protein and autoinducer-2; ribose binding protein acts as an antagonist on the receptors of autoinducer-2 [95]. Brominated furanones have a negative influence on biofilms of *P. gingivalis*, although they do not affect the growth *of P. gingivalis* [94]. Studies have shown that acylase, lactonase and oxyreductase are potential quorum quenching agents that inactivate the acyl-homoserine lactone, potentially affecting periodontal pathogens [96]. Thus, autoinducer-2 inhibition is an attractive therapeutic intervention target for treating periodontal diseases.

### Immune modulation as a therapeutic approach for periodontal disease

Recently, immunomodulatory therapy that regulates the immune response has received much attention. Immunomodulatory therapy is a promising option for treating periodontal diseases [97]. Immunomodulators help to reduce bone loss by controlling the osteolytic and inflammatory process [98], preventing or intervening in the progression of periodontal disease.

The immune microenvironment of periodontitis-related tissue is critically essential for periodontal disease. Increased infiltration of leukocytes and the release of inflammatory molecules can resolve inflammation effectively. However, excessive amounts of this inflammatory response can cause serious harm to the periodontal tissues and the alveolar bone. Modulation of this microenvironment can not only supplement traditional treatments for periodontal disease but also may promote periodontal regeneration [97]. Different approaches have been taken to modulate leukocytes and the other inflammatory cytokines in the microenvironment, including immunomodulatory drug therapy, stem cell therapy, and gene therapy.

Among the leukocytes, macrophages play a significant role in various stages of periodontitis [99]. Neutrophils are key players in maintaining the homeostasis of periodontal health and are considered the first line of defence against periodontitis [98, 100, 101]. Monocytes also contribute to the defence system, and monocyte cells present in higher numbers in the intermediate stage of periodontal disease [102]. At the same time, T lymphocytes can play a vital role in gingival health and alveolar bone resorption [103–105].

Drug therapy targeting immune response is promising for treating periodontal diseases. Resveratrol is a natural polyphenol and can inhibit NF-kB activation in macrophages, and it shows immunomodulatory effects on *F. nucleatum* by upregulation of the antioxidant pathway [106, 107]. Metformin can inhibit the production of nitric oxide synthase in monocytes, affecting periodontitis [108]. Metformin reduces inflammation by the regulation of IL-1β [109]. Catechin is another polyphenol used as an immunomodulator. In the mice model, catechin is effective against gingivitis by reducing the lL-1β in the macrophage [110]. Gliclazide has antioxidant properties and can reduce the infiltration of neutrophils and macrophages, as shown in the rat model with periodontal disease [111]. Moreover, it can reduce inflammation by decreasing the level of TNF-α [111].

Recent studies have shown that curcumin can release pro-inflammatory molecules, which is effective against periodontal pathogens [112]. CMC2.24 is a modified version of curcumin that reduces phagocytic activity in macrophages, and its anti-inflammatory properties have been shown against periodontal disease [113, 114].

Trans-cinnamic aldehyde is also used as a drug for treating *A. actinomycetemcomitans,* which involves the downregulation of TNF-α and IL-1β cytokines [115]. Kava-205Me lowers the level of IFN-γ and IL-12 and is effective against *P. gingivalis*. Other drug therapy includes carnosic acid, glyburide, and bismuth drugs, which show their immunomodulation by infiltrating inflammatory cells and reducing pro-inflammatory cytokines such as IL-6, IL-1β and TNF-α [116, 117].

Immuno-related gene therapy is another novel approach targeting host immunity. It has been shown that downregulation of T-cell immune response cDNA7 (TIRC7) leads to a lower number of T-cells in gingival tissue, which affects the periodontal disease [118]. Plasmid DNA encoding miR-200c injected into gingiva has been shown to prevent gingival inflammation [119]. Cathepsin K (Ctsk) can reduce inflammation and osteoclast activity by lowering the level of TNF-α, INF-γ, IL-1α, IL-1β, and IL-12, thus regulating periodontal health [120]. P2X7 receptor (P2X7R) can modify the local microenvironment of periodontitis and improve the bone tissue regeneration [121]. The mode of action of TNF receptor and immunoglobulin Fc (TNFR: Fc) involves infiltration of leukocytes and reduction of cytokines, notably IL-1β, TNF-α, IL-6 and IL-10 [122].

Stem cell therapy has significant potential to impact periodontal health. Different mesenchymal stem cells can modulate the microenvironment coupled with the inflammatory response of leukocytes and cytokines associated with periodontal disease. These stem cells are being explored as a promising therapeutic intervention for periodontal disease. Periodontal ligament stem cells (PDLSCs) can interact with immune cells by modulating their activities and thus work as a potential therapeutic approach for treating periodontal diseases [123]. PDLSc can enhance tissue regeneration by converting macrophages to anti-inflammatory phenotypes and suppress the immune response by inactivating B-cells [124, 125]. PDLSc can be drastically elevated with resveratrol treatment. In periodontal patients, resveratrol treatment partially decreases bone loss and inhibits the infiltration of T-cells [126]. Gingiva-Derived Mesenchymal Stem cells (GMSCs) promote the polarization of M1 to M2 macrophages, lower the infiltration of neutrophils and decrease pro-inflammatory cytokines [127–129]. Mesenchymal stem cells derived from human exfoliated deciduous teeth showed reduced levels of TNF-α, IFN-γ and IL-2 and promoted anti-inflammatory response in macrophages [130, 131]. Dental Follicle Stem Cells (DFSCs) regulate peripheral blood mononuclear cells by enhancing the levels of IL-10 but lowering the levels of IFN-γ and IL-4 and can thus play an essential role as immunomodulators in the treatment of periodontal diseases [132, 133]. Bone marrow mesenchymal stem cells and dental pulp stem cells (DPSCs) decrease the level of TNF-α, IFN-γ and IL-17 in periodontal pathogens [131, 134, 135]. In addition, DPSCs can regenerate different tissue, which includes bone, and those cells are easy to store [136]. Thus, these approaches are getting much attention for treating periodontal diseases.

Low dose antibiotics are another group of agents that can modulate host immune response, potentially contributing to the periodontal disease progression or resolution. Doxycycline at a low dose, i.e. at sub-inhibitory concentrations, inhibits matrix metalloproteinase‐8 (MMP-8), which is present abundantly in periodontitis [137]. A pilot study by Ryan et al. investigated chemically modified tetracycline-3 in periodontal patients receiving scaling and root planing. Administration of this antimicrobial agent at a low dose (10 mg/d) reduced the levels of interleukin‐1-beta and matrix metalloproteinase‐8 in gingival crevicular fluid moderately [138]. Several other studies have shown reduce pocket depth (PD) and gain in clinical attachment level (CAL) in patients with periodontal disease when sub-antimicrobial dose of doxycycline was administrated [139, 140]. Also, studies reported significantly improved clinical parameters and reduced gingival inflammation in the host [139, 141, 142].

Table 4 lists the recent studies in the literature in which the immune microenvironment is targeted, together with the cytokines and leukocyte targets of these immune therapies.

**Table 4:** A summary of immunotherapies and their targets

Type of Therapy		Immunomodulatory activity on targets			References
		**Monocytes/Macrophage**	**Lymphocyte**	**Cytokines**	
**Drug therapy**	**Resveratrol**	Treatment of gingival keratinocytes with Resveratrol inhibited NF-kB activation and reduced the marker TREM-1 in monocytes	/	/	[107]
	**Metformin**	In Monocytes, metformin hindered the production of nitric oxide synthase and nitric oxide induced with LPS. In addition, this drug decreased the expression of chemokine (C–C motif) ligand 2 (CCL-2) to improve inflammation	/	Delivery of metformin in diabetic mice with periodontitis effectively decreased NLRP3 and IL-1β levels reducing inflammation	[108, 109]
	**Catechin**	/	/	Catechin decreased IL-1β level from macrophages in mice model of gingivitis	[110]
	**Gliclazide**	Gliclazide treatment in a rat model of periodontitis reduced infiltration of neutrophils, macrophages and reduced activity of myeloperoxidase enzyme	/	This drug also reduced inflammation by declining the levels of IL-1β and TNF-α, as well as reduced the expression of COX-2 and cathepsin k	[111]
	**Curcumin (CMC2.24)**	Chemically-Modified version of Curcumin (CMC2.24) reduced phagocytic activity of macrophages	/	Also, curcumin reduced oxidative stress, bone resorption and inflammatory response by decreasing the levels of TNF-α, IL-1β, IL-10	[113, 199]
	**Trans-cinnamic aldehyde**	/	/	*A. actinomycetemcomitans* infected cells were pre-treated with trans-cinnamic aldehyde which lowered the levels of TNF-α, IL-1β	[115]
	**The Amyl-1–18 peptide**	This peptide was derived from rice which can inhibit the secretion of IL-6 from macrophage		This peptide can also counteract LPS, suppress signal transduction in NF-kB as well as IL-1R signaling pathway	[200]
	**Kava-205Me**	/	/	Addition of methyl group to natural compound kavain is known as Kava-205Me (5,5-dimethyl-3-oxocyclohex-1-en-1-yl 4-methylbenzoate). Kavain is generally obtained from *Piper methysticum* Human cell lines were infected with *P. gingivalis* and treated with Kava-205Me at different doses. This compound could decrease the levels of IL-12, IL-10, IFN-γ and exotoxin. These cytokines indicate the early phase of inflammation	[201]
	**Carnosic Acid**	Carnosic acid reduced infiltration of inflammatory cells	/	This phenolic diterpene which is a natural compound imparted anti-oxidative and anti-inflammatory properties in mice model challenged with LPS. Application of carnosic acid led to the decrease in expression of CXCL9, CXCL10, and CXCL11	[202]
	**Glyburide**	Glyburide is a blood sugar lowering drug. Treatment with glyburide declines infiltration of inflammatory cells and number of osteoclast cells	/	THP-1 which is a macrophage-like cell line was stimulated with periodontal pathogenic bacteria and treated with glyburide, which resulted in decrease of IL-1β levels in culture media but increase in expression of endogenous IL-1β	[116]
	**Bismuth drugs**	/	/	Treatment of *P. gingivalis* challenged human cells with bismuth drugs decreased its iron acquisition ability and reduced the levels of proinflammatory cytokines such as IL-6, IL-1β and TNF-α	[117]
**Gene therapy**	**T-cell immune response cDNA7 (TIRC7)** Isoform of Atp6i	/	Downregulation of TIRC7 was achieved by shRNA and delivered locally by Adeno-Associated Virus (AAV). This modification of gene led to the lowering T cells in the gingival tissue	In addition, silencing of TIRC7 triggers the reduction in levels of IL-6, IL-17A and Cathepsin K (Ctsk) in periodontal tissue	[118]
	**MicroRNA-200c (miR-200c)**	/	/	Plasmid DNA encoding miR-200c was directly injected into the gingiva which rescued the downregulation of miR-200c as well as prevented periodontal inflammation. In addition, miR-200c effectively decreased the levels of IL-6 and IL-8 at transcription level which is involved in sustaining systemic inflammation in periodontitis	[119]
	**Ctsk**	/	Ctsk was downregulated by shRNA and carried in AAV to deliver in periodontal ligament cells, which reduced the number of T cells and dendritic cells. This abolished inflammation and osteoclast activity	Silencing of Ctsk also decreased the levels of cytokines such as TNF-α, INF-γ, IL-1α, IL-1β, IL-12 and IL-17 while upregulates the levels of IL-6 in vivo	[120]
	**P2X7R**	/	/	P2X7 receptor (P2X7R) gene-modified stem cells help to reduce inflammation. Moreover, this genetically modified cells ameliorate the harsh local microenvironment of periodontitis through secreting molecules on neighboring cells and improve tissue regeneration	[121]
	**TNFR: Fc**	/	In rat model of periodontal disease, application of a fusion gene containing TNF receptor and immunoglobulin Fc (TNFR: Fc) by AAV increase the expression of TNFR protein in serum at therapeutic level. This protein also reduced infiltration of leukocytes at the site of infection	Moreover, TNFR protein decreased the levels of several cytokines such as IL-1β, TNF-α, IL-6 and IL-10 which reduced inflammation as well as prevented bone loss	[122]
**Stem cell therapy**	**Periodontal ligament stem cells (PDLSCs)**	Application of PDLSCs promote the conversion of macrophages to the anti-inflammatory (M2) phenotype which helps to enhanced regeneration of tissues	PDLSCs can suppress immune response by performing anergy of T-cell and decreasing the proliferation, differentiation and activation of B cells	Moreover, a significant amount of decrease in the level of glycoprotein named CD1b was observed on dendritic cells due to the application of PDLSCs. This stem cell therapy inhibited the proliferation of T cell suggesting a promising a novel therapeutic approach	[124, 125, 203]
	**Gingiva-Derived Mesenchymal Stem cells (GMSCs)**	Extracellular vesicles obtained from GMSC promote the polarization of M1 to M2 macrophages	GMSCs can reduce the infiltration of neutrophils, Th17 cells, as well as increase the migration of Tregs	Application of GMSCs decreased pro-inflammatory cytokines such as TNF-α, IL-1β and IL-6 significantly while increases the anti-inflammatory cytokine IL-10	[108, 127, 128]
	**Mesenchymal stem cells derived from human exfoliated deciduous teeth (SHEDs)**	Enhance the differentiation of anti-inflammatory macrophages	Promote an immunomodulatory function in monocyte derived from dendritic cells	Application of SHED at different dose showed a decrease in levels of TNF-α, IFN-γ and IL-2, and an increase in IL-10 showed in rat model	[130, 131, 204]
	**Dental Follicle Stem Cells (DFSCs)**	/	Increased the number of Tregs	When stimulated by IFN-γ, DFSCs can modulate peripheral blood mononuclear cells (PBMCs) obtained from healthy donor by enhancing the levels of IL-10 but decreasing the levels of IFN-γ and IL-4 DFSCs showed immune response to lipopolysaccharide (LPS) from *P. gingivalis* leading to decreased levels of TLR4 mRNA significantly. This was accompanied by an increased migration of the stem cells without altering the levels of IL-6	[132, 133]
	**Bone Marrow Mesenchymal Stem Cells (BMMSCs)**	Extracellular vesicles derived from BMMSCs decreased the levels of M1 macrophages and increased the levels of M2 macrophages	/	In periodontitis model of rat, transplantation of Acetylsalicylic acid treated BMMSCs increased IL-10 and reduced tumor necrosis factor-α (TNF-α) and IL-17 reducing inflammation In another study of rat model with periodontal disease, application of BMMScs showed reduction in levels of IL-1β, TNF-α,IL-17	[205–207] [134, 208]
	**Dental pulp stem cells (DPSCs)**	Moreover, exosomes-incorporated in chitosan hydrogel obtained from DPSCs enhanced the conversion of proinflammatory state macrophages to an anti-inflammatory state	Increased the number of Tregs and decreased Th17 cells	LPS challenged co-culture of DPSCs and PBMCs showed decrease in TNF-α, IFN-γ, IL-2, IL-17 and an increase in IL-10 secretion without altering the levels of IL-6 and IL-1β	[131, 135, 209, 210]
**Other therapy**	**Photodynamic** **therapy (PDT)**	/	/	Decreased the levels of TNF-α in asthmatic animals challenged with *P. gingivalis* Decreased the level of IL-6, IL-8, and CXCL10 in *A. actinomycetemcomitans* and promotes periodontal regeneration	[211] [212]
	**Methylene blue-mediated photodynamic therapy (MBPDT)**	Induction of apoptosis in macrophage due to oxidative stress as well as mitochondria dependent apoptosis pathways were shown in vitro	/	Lower levels of IL-1β and TNF-α was observed	[213]
	**Low-intensity pulsed ultrasound (LIPUS)**	/	/	Treatment suppressed a range of cytokines at transcription level including IL-1a, IL-1b, IL-6, IL-8 as well as reduced the levels of IL-1ß and tumor necrosis factor a (TNFα) by periodontal ligament fibroblasts (PDLFs)	[214]
	**Singlet** **phototherapy**	Infiltration of macrophages in periodontitis and significant vascularization of periodontal tissue that contributed to tissue regeneration and ameliorate infection	/	/	[215]
	**Indocyanine green (ICG)-diode laser-based photothermal** **therapy (PTT)**	/	/	Reduction of IL-1β and MMP-8 at 6 months after treatment in patients with gingival inflammation	[213]

### Modification of periodontal microbiota by probiotic and microbiota replacement therapy

In contrast to the traditional view of individual pathogens being responsible for disease onset, the new perspective of periodontal disease deems that the transition from health to disease status is attributed to a shift in the global balance of the microbial flora (i.e. disruption of microbial homeostasis). Periodontal disease is considered a result of disrupted microbial homeostasis and microbe-mediated disruption of host homeostasis. Such a perspective has opened new paths for periodontal disease treatment, including restoring the homeostasis of the microbiota associated with periodontal health.

Recent studies have suggested probiotic therapy for treating periodontal diseases in the oral cavity [143, 144]. Probiotics help control diseases presumably through immune modulation and colonization resistance to pathogens. The selection of the microbial strain is crucial for the treatment outcome of the probiotic therapy. Most of the chosen probiotics originated from gut microbiota or fermented foods [145]. Recent clinical trials have shown that the administration of probiotics improves bacterial dysbiosis in periodontal patients [146]. Beneficial bacteria create a substantial barrier against colonization of endogenous and exogenous pathogens [8]. Antagonistic bacteria of probiotics have the potential to fight against periodontal bacteria [37]. The study by Van et al. has shown that clinical isolates collected from healthy patients significantly inhibited the growth of *P. intermedia* or *P. gingivalis,* which was high in the diseased patient sample [147]. These pathogen-inhibiting isolates include *Bifidobacterium, Streptococcus,* and *Actinomyces*. Commercially available dietary probiotics have been shown to have more substantial inhibitory properties against periodontal bacteria [8]. A successful periodontal therapeutic approach is characterized by the alleviation of periodontal inflammation, which relates to changes in microflora, and in that aspect, probiotic therapy may potentially plays a role [148].

A study comparing the prevalence of oral Lactobacilli in healthy subjects and patients with periodontitis (chronic) showed that *Lactobacillus fermentum* and *Lactobacillus gasseri* were the most common species in healthy subjects. In contrast, *Lactobacillus plantarum* was the most prevalent species in patients with periodontitis [149]. In addition, four species of Lactobacilli have shown to have the greatest antimicrobial properties, which is confirmed by international guidelines for probiotics evaluation. Those isolates include *Lactobacillus salivarius, Lactobacillus plantarum*, *Lactobacillus rhamnosus* and *Lactobacillus paracasei*, which can be used as a probiotics for maintaining oral health [150]. A report showed that *Lactobacillus reuteri* could be as efficacious as probiotics for reducing dental-biofilm induced by systemic or local risk factors [151]. Studies of fifty-one patients showed yogurt supplemented with *Bifidobacterium animalis* significantly impacted inflammatory parameters of gingiva and bacterial plaque [152].

Microbiota replacement therapy is a new disease treatment/prevention measure in which microbiota from healthy donors is transplanted to diseased patients. Replacement therapies have been successfully used to treat *Clostridium difficile* infections. Also, it has shown efficacy for treating one class of inflammatory bowel diseases known as Crohn’s disease. [153, 154]. Microbiota replacement therapies are currently being explored as an alternative approach to treating periodontal disease [37]. Naturally occurring and laboratory-derived oral bacteria can be used in replacement therapy of periodontal diseases. Protocols have been suggested for transferring microbiota from a healthy donor to a patient with periodontal diseases [155, 156]. Recent study protocol has shown that oral microbiota transplant therapy could effectively treat periodontal disease by modulating the oral microbiota. For oral microbiota transplant therapy, details of the donor's medical history and microbiota analysis are essential [157]. With the advancement of the human microbiome and a better understanding of the relationship between oral microbiota and oral diseases, it is expected that more practical and more effective microbiota replacement therapy approaches for periodontal diseases will be available in the future.

## Conclusion

Although anti-virulence therapeutic strategies are relatively new in preventing and treating periodontal disease, novel drugs targeting the critical virulence factors of periodontal pathogens have already been identified, which serve as a promising alternative in the era of antibiotic resistance. A growing library of novel targets will open the door to rapidly identifying anti-virulence drugs.

Among the approaches that target specific virulence factors to treat periodontal diseases, protease inhibitors against gingipains have been tested in vivo. Although receptor-based peptide inhibitors against LtxA activity and peptides interfering with fimbrial biogenesis show significant results, animal models' potency must be tested to validate their effectiveness.

The formation of biofilm by oral pathogens makes these pathogens challenging to treat with antibiotics. Compounds with bioactive in quorum quenching and downregulating adherence factors in microbes can be used to prevent or clear up microbial biofilms. Further studies are required to test the quorum quenching strategies to combat biofilm formation by periodontal pathogens.

Plant-derived natural bioactive compounds impart general inhibitory effects on many oral pathogens. The growing emergence and spread of antimicrobial resistance call for renewed interests in plant-based compounds such as polyphenols, which have few side effects to the host. Antimicrobial activity and anti-inflammatory effects of these natural compounds make them an attractive remedy for periodontal diseases. However, the effective doses of these natural antimicrobial compounds need to be determined and their antimicrobial mechanisms assessed before they can be used in conjunction with traditional treatment approaches.

Immunomodulatory therapy and microbiome-based therapies show exciting promises in treating periodontal diseases. Still, further research is needed to improve their efficacy and to understand their long-term effects.

In summary, significant progress has been made in our understanding of periodontal disease and its treatment strategies in recent years. We can expect novel approaches will become available for periodontal disease prevention and treatment in the near future.

## Data Availability

Data sharing does not apply to this article as no datasets were generated or analyzed during the current study.

## Abbreviations

AAP: American Academy of Periodontology
EFP: European Federation of Periodontology
EPS: Extracellular polymeric substances
CPS: Capsular polysaccharide
LPS: Lipopolysaccharide
IL: Interleukin
LtxA: Leukotoxin
CDT: Cytolethal distending toxin
LFA-1: Lymphocyte function-associated antigen-1
OMVs: Outer membrane vesicle
DANA: 2-Deoxy-2,3-didehydro-N-acetylneuraminic acid
EGCG: Epigallocatechin gallate
QS: Quorum sensing
TIRC7: T-cell immune response cDNA7
Ctsk: Cathepsin K
TNFR: Fc: TNF receptor and immunoglobulin Fc
PDLSCs: Periodontal ligament stem cells
GMSCs: Gingiva-derived mesenchymal stem cells
DFSCs: Dental follicle stem cells
DPSCs: Dental pulp stem cells
MMP-8: Matrix metalloproteinase‐8
PD: Pocket depth
CAL: Clinical attachment level
PCL: Polycaprolactone
PI: Plaque index
GI: Gingival index
PEG: Poly Ethylene Glycol
TCS: Thiolated Chitosan
PMAA: Poly (methacrylic acid)
PDLGA: Poly Lactic-Co-glycolic acid
PD: Probing depth
AAV: Adeno-associated virus
P2X7R: P2X7 receptor
TNF-α: Tumor necrosis factor-α
PDT: Photodynamic therapy
